# Curcumin Incorporated Poly(Butylene Adipate-co-Terephthalate) Film with Improved Water Vapor Barrier and Antioxidant Properties

**DOI:** 10.3390/ma13194369

**Published:** 2020-09-30

**Authors:** Swarup Roy, Jong-Whan Rhim

**Affiliations:** Department of Food and Nutrition, BioNanocomposite Research Institute, Kyung Hee University, 26 Kyungheedae-ro, Dongdaemun-gu, Seoul 02447, Korea; swaruproy2013@gmail.com

**Keywords:** PBAT, curcumin, bioactive functional film, water vapor barrier property, antioxidant activity

## Abstract

Curcumin incorporated poly(butylene adipate-co-terephthalate) (PBAT) based film was fabricated. Curcumin has uniformly distributed in the PBAT matrix to form a bright yellow PBAT/curcumin film. The PBAT/curcumin film has slightly reduced tensile strength and flexibility than the neat PBAT film, while the thermal stability of the film has not changed significantly. The blending of curcumin significantly decreased the water vapor permeability of the PBAT film. Additionally, the PBAT/curcumin film showed potent antioxidant activity with some antimicrobial activity. The PBAT/curcumin films with improved water vapor barrier and additional functions can be used for active packaging applications.

## 1. Introduction

Petrochemical-based synthetic plastics gain popularity among all packaging materials due to their lightness, cost-effectiveness, user-friendliness, corrosion resistance, and structural properties [1]. Among the synthetic plastics commonly used in various packaging products are polypropylene, polystyrene, high and low-density polyethylene, polyethylene terephthalate, and polyvinyl chloride. [2]. Plastics production worldwide has reached ~450 million tons each year, of which about 40% is used in the packaging industry [2]. The increase in plastics production is very alarming, and the growth rate of plastics production worldwide is expected to reach ~1800 million tons in 2050 [3]. Currently, there is an increasing demand for eco-friendly, biodegradable, and renewable plastics because too many non-biodegradable plastic packaging materials are generated, which can not only affect the environment and eco-systems but also cause serious plastic contamination [2,4,5]. Therefore, biodegradable and bio-based plastic packaging materials have gained significant attention for substituting non-biodegradable petroleum-based plastic polymers [6,7,8]. Biodegradable plastics are much more environmentally-friendly than petroleum-based plastics because they are easily decomposed by microorganisms in the environment, producing carbon dioxide and water [9,10]. Polyesters such as PBAT (poly(butylene adipate-co-terephthalate)), PLA (poly(lactide)), and PHA (poly(hydroxy alkanoates)) are of great interest due to their biodegradability and hydrolysis of ester bonds to non-toxic substances [11,12]. Among these polyesters, PBAT is considered one of the most likely biodegradable polymers for food packaging film applications [13,14,15]. PBAT is an aliphatic, aromatic polyester, mainly obtained from 1,4-butanediol, adipic acid, and terephthalic acid. PBAT is readily dissolved in chloroform, making it possible to prepare PBAT film using a simple solution casting technique. The aliphatic portion of PBAT provides good biodegradability, and the aromatic portion provides good mechanical properties [16]. PBAT is also very flexible, which is useful for flexible packaging applications [17]. Despite the useful functional properties of PBAT, its use is sometimes limited due to its weak barrier properties [9,17]. Therefore, it is necessary to expand the properties of the PBAT film to replace the petroleum-based polymers currently used in the packaging area. A variety of functional or reinforcing fillers such as SiO_2_, graphene oxide, MgO, AgNP, ZnONP, clay, curcumin, grape seed extract, etc. have been utilized enhance the physical and functional properties of PBAT films [9,15,17,18,19,20,21,22,23,24,25]. In this sense, the use of natural bioactive compounds to improve PBAT film properties is an attractive field of research.

Curcumin, a natural bioactive compound, is interesting because it is non-toxic and shows numerous benefits and applications that are already very well known [26,27,28]. Curcumin is a hydrophobic phenolic substance derived from *Curcumin longa*, also known as turmeric, and has long been used as a spice in food and medicine to treat various diseases [28]. Although curcumin has high clinical applicability, its low water solubility, absorbability, and metabolism have limited its direct use in biomedicine [29]. Curcumin has been used to produce functional films mixed with various polymers such as PLA, PBAT, LDPE, cellulose, pectin, carrageenan, gelatin, etc. for biomedical and food packaging applications [21,30,31,32,33,34,35,36,37,38,39]. Recently, De Compos et al. reported the preparation of curcumin-reinforced PBAT and thermoplastic cassava starch-based extruded composite films [21]. In addition, PBAT-based electrospun nanofiber has been developed by reinforcing curcumin and 5-fluorouracil [39]. As far as we know, there are no reports of concentration-dependent curcumin effects on the film properties of PBAT-based films using the solution casting method for active packaging applications. The solution casting method is simple and useful for testing the effectiveness of fillers and determining laboratory-scale processing conditions. The information obtained can be used for large-scale production using the extrusion method. Curcumin is a useful bioactive natural functional compound used very recently to make functional films compounded with various bioplastics. PBAT is the right candidate for making functional packaging film compounded with curcumin. Since both PBAT and curcumin are soluble in the same solvent (chloroform), the solution casting method is the right choice for preparing the PBAT/curcumin blending film. The addition of curcumin to PBAT-based film is likely to improve the physical and functional properties of the film.

The main objective of the present work was to prepare a functional PBAT-based film by mixing with curcumin. The bioactive film was characterized using various analytical methods. Additionally, the effect of various concentrations of curcumin on different physical (surface color, optical, mechanical, and water vapor barrier properties) and functional properties (antioxidant and antimicrobial) and the release profile of curcumin from the PBAT-based films were investigated.

## 2. Materials and Methods

### 2.1. Materials

PBAT (EnPol PBG7070; m.p. 125 °C, the specific gravity of 1.20–1.25) was acquired from S-EnPol Co. Ltd., (Wonju, Korea). Curcumin, DPPH, ABTS, and potassium persulfate were purchased from Sigma-Aldrich, (St. Louis, MO, USA). Chloroform and ascorbic acid were procured from Daejung Chemicals and Metals Co., Ltd., (Siheung, Korea). TSB, BHI media, and agar powder were purchased from Duksan Pure Chemicals Co., Ltd., (Ansan, Korea). *Escherichia coli* O157: H7 ATCC 43895 and *Listeria monocytogenes* ATCC 15313 were acquired from the Korean Collection for Type Culture (KCTC, Seoul, Korea).

### 2.2. Fabrication of PBAT/Curcumin Films

The PBAT/curcumin films were fabricated using a solution casting method [19]. Various amounts of curcumin (0.125, 0.25, 0.5, and 1.0 wt% of PBAT) were dissolved in 100 mL of chloroform with stirring for 1 h and added 4 g of PBAT to the curcumin solution and mixed with vigorous stirring for 24 h. The film solution was then cast on a leveled Teflon film-coated glass plate and evaporated the solvent in a fume hood for 48 h. Then the dried film was peeled off the plate and conditioned for at least 48 h in a humidity chamber controlled at 25 °C and 50% RH. For comparison, a neat PBAT film without curcumin was also produced according to the prescribed method. All films were made in triplicate and used as experimental replication units. The prepared films were designated PBAT/cur^0.125^, PBAT/Cur^0.25^, PBAT/Cur^0.5^, and PBAT/Cur^1.0^, respectively, according to the curcumin content.

### 2.3. Characterization and Properties of PBAT/Curcumin Film

#### 2.3.1. Surface Morphology

The microstructure (surface and cross-section) of the film was observed using a field emission scanning electron microscope (FE-SEM, SU-8000, Hitachi Co., Ltd., Matsuda, Japan).

#### 2.3.2. Thermal Analysis

The thermal stability of the film and curcumin was tested using a thermogravimetric analyzer (Hi-Res TGA 2950, TA Instrument, New Castle, DE, USA) following the method of Roy et al. [40]. The temperature of the maximum disintegration rate was determined from a derivative form of the TGA (DTG) curve, and the weight loss (%) was calculated from the TGA curve [40].

The melt-crystallization of the film samples was evaluated using a differential scanning calorimeter (DSCQ100, TA Instruments, New Castle, DE, USA), and the transition temperature was calculated from the DSC thermogram following the method of Sousa et al. [41].

#### 2.3.3. Surface Color and Optical Properties

The surface color of the film samples was assessed using a Chroma meter (Konica Minolta, CR-400, Tokyo, Japan). The Hunter color (*L*, *a*, *b*) of the film sample was obtained, and the total color difference (Δ*E*) was computed using Equation (1).

Δ*E* = [(Δ*L*)^2^ + (Δ*a*)^2^ + (Δ*b*)^2^]^0.5^ (1)

where Δ*L*, Δ*a*, and Δ*b* is the difference of each color values between the standard color plate and film sample, respectively. The yellowness index (*YI*) of the film was determined using Equation (2).

*YI* = (142.86 × *b*)/*L* (2)

The optical properties of the films were evaluated using a UV-vis spectrophotometer (Mecasys Optizen POP Series UV/Vis, Seoul, Korea). The light absorbance of the film was taken in the wavelength range of 200 nm–800 nm. UV-barrier and transparency properties of the film samples were assessed by measuring the light transmittance at 280 nm (T_280_) and 660 nm (T_660_), respectively [34].

#### 2.3.4. Mechanical Properties

A digital micrometer (QuantuMike IP 65, Mitutoyo, Japan) with an accuracy of 1 μm was used to measure the thickness of the film samples [40].

The mechanical properties were measured using an Instron Universal Testing Machine (Model 5565, Instron Engineering Corporation, Canton, MA, USA) following the standard ASTM method D 882-88. The machine was operated with an initial grip separation of 50 mm and a crosshead speed of 50 mm/min using a 500 N load cell [27].

#### 2.3.5. Water Vapor Permeability (WVP) and Water Contact Angle (WCA)

The water vapor permeability (WVP) of the film sample was measured at 25 °C under 50% RH conditions [27]. First, the water vapor transmission rate (WVTR, g/m^2^ s) was determined according to the ASTM E96-95 method. For this, 18 mL of distilled water was added to the WVP cup (6.8 cm diameter and 2.5 cm depth) made of poly(methylmethacrylate), fixed the film sample on the cup, and sealed tightly. The assembled WVP cup was put in a humidity chamber (25 °C and 50% RH), and measured the weight change periodically. The WVTR of the film was calculated from the slope of the weight change of the WVP cup vs. time curve, and the WVP (g m/m^2^ Pa.s) of the film was computed using Equation (3).

WVP = (WVTR × L)/∆p(3)

where L was the mean film thickness (m), and ∆p was the partial water vapor pressure difference across the two sides of the film [42].

The water contact angle of the film was determined using the water contact angle (WCA) analyzer (Phoneix 150, Surface Electro Optics Co., Ltd., Kunpo, Korea). For this, the film sample was positioned on the WCA analyzer, and 10 μL of water drop was added to the film and measured the WCA immediately [43].

### 2.4. Curcumin Releasing Test

The quantity of curcumin released from the PBAT/curcumin film into the water was determined following Roy and Rhim [27]. For this, the test film (PBAT/Cur^0.25^ and PBAT/Cur^1.0^) samples (2.5 cm × 2.5 cm) were transferred in 20 mL of distilled water and incubated at 37 °C. A 2 mL solution sample was taken at predetermined intervals and measured the absorbance of the sample at 420 nm.

### 2.5. Antibacterial Activity

The antibacterial activity of the film was evaluated against foodborne pathogenic bacteria, *E. coli* and *L. monocytogenes* [34]. The test bacteria were first aseptically inoculated into TSB and BHI broth, respectively, and incubated at 37 °C for 24 h. After properly diluting the culture, 200 μL of the diluted inoculum (10^8^–10^9^ CFU/mL) was aseptically transferred to 50 mL of TSB and BHI broth, respectively, together with 200 mg of the film samples to reach the initial concentration of bacteria around 10^6^ CFU/mL, and incubated at 37 °C for 12 h with shaking at 100 rpm. Samples were taken at predetermined intervals, and the number of viable cells was measured by diluting and plating samples on agar plates.

### 2.6. Antioxidant Activity

The antioxidant activity of curcumin and PBAT/curcumin film samples was evaluated using DPPH^•^ and ABTS^•+^ free radical scavenging activities [33,44]. For the DPPH assay of the PBAT/curcumin film, a predetermined amount of the films were added to 10 mL DPPH solution and measured the absorbance at 517 nm after 24 h. For the ABTS analysis, a predetermined amount of the test films were mixed with 10 mL of ABTS assay solution and measured the absorbance at 734 nm after 24 h. The antioxidative activity of the films was calculated using Equation (4):

(4) $$\mathrm{Free}\;\mathrm{radical}\;\mathrm{scavenging}\;\mathrm{activity}\;\left(\%\right)=\;\frac{A_{0}-A_{T}}{A_{0}}\times 100\;\;\;\;\;\;\;\;\;\;\;\;$$

where *A*_0_ and *A*_T_ was the absorbance of DPPH or ABTS of the control and test film, respectively. All the test was performed in triplicate, and the average value was reported.

### 2.7. Statistical Analysis

Film properties were measured in triplicates of separately prepared films, and the results were presented as mean ± SD (standard deviation). ANOVA test was performed, and the significant difference (*p* < 0.05) among treatment groups was separated by Duncan’s multiple range test using the SPSS computer program (SPSS Inc., Chicago, IL, USA).

## 3. Results and Discussion

### 3.1. Morphology

**Figure 1:**
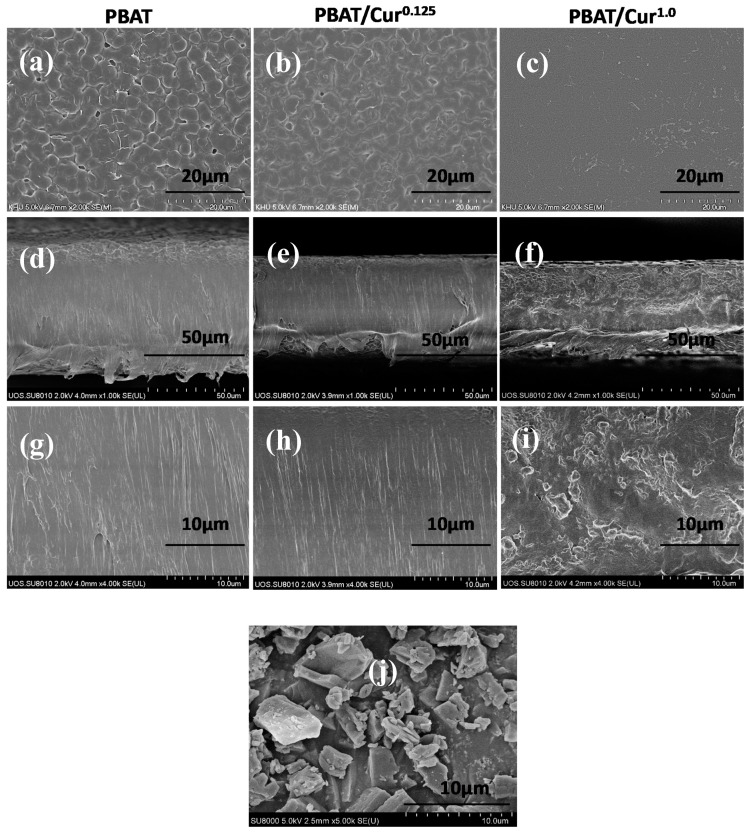
FE-SEM pictures of surface (**a**–**c**) and cross-section (**d**–**h**) of the neat poly(butylene adipate-co-terephthalate) (PBAT) and PBAT/curcumin films (**d**–**f**: low and **g**–**i**: high mnagnification) and FE-SEM image of curcumin powder (**j**).

All films were smooth and flexible without any defects. The surface microstructure of the neat PBAT and PBAT/curcumin films is shown in Figure 1a–c. It showed the smooth-surfaced films, in which curcumin was evenly dispersed in the PBAT matrix. At a small amount of curcumin, it formed a compatible film with the PBAT without creating an aggregation of the particle. Even at high curcumin (1 wt%), the film surface was smooth without any cavity between curcumin and PBAT polymer. The cross-sectional FESEM images (Figure 1d–i) also showed well-dispersed curcumin in the PBAT matrix when the curcumin content was low, while curcumin was aggregated when a higher content was added. The morphology of the films designated that the curcumin was blended compatibly with the PBAT matrix. Similarly, curcumin was reported to be well dispersed in LDPE and cellulose-based films [31,45]. The FESEM image of curcumin (Figure 1j) showed that curcumin has an asymmetrical form that complies with previously published reports [34].

### 3.2. Thermostability

**Figure 2:**
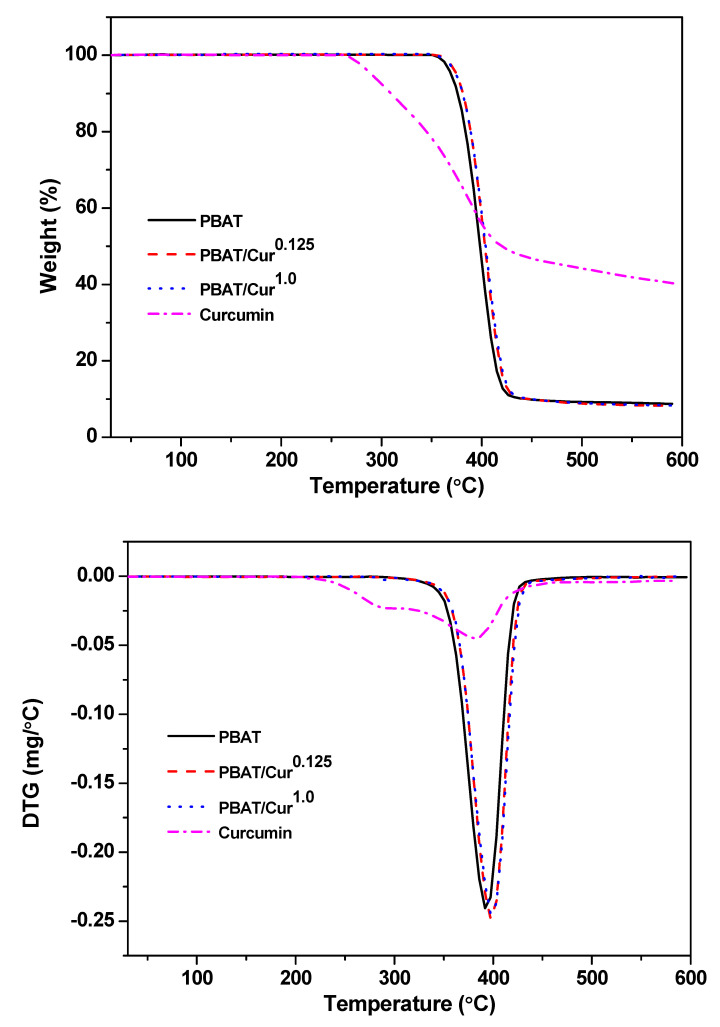
TGA and DTG thermograms of the neat PBAT and PBAT/curcumin films and curcumin powder.

Figure 2 shows the TGA and DTG thermograms of the neat PBAT, PBAT/curcumin films with low (0.125 wt%) and high content (1.0 wt%) of curcumin and curcumin powder. All films exhibited one-step thermal decomposition at 350 °C–430 °C with maximal degradation at 398 °C. Similar thermal degradation patterns were observed in PBAT/silver nanoparticle films [19]. The remaining char content of the PBAT film was ~8% and enhanced a little after mixing with curcumin. In curcumin powder, the thermal decomposition pattern was different from the PBAT film, although the maximum degradation temperature was in a similar temperature range. The analysis data of the thermal degradation of PBAT/curcumin films and curcumin powder were shown in Table 1. The TGA test results showed that the thermal stability PBAT was not affected by the blending of curcumin. Current results were also consistent with previously published reports [34].

**Figure 3:**
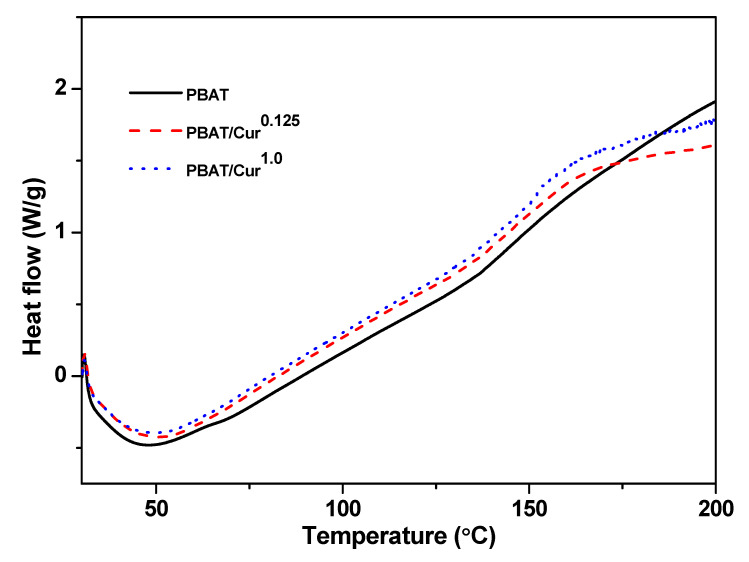
DSC thermogram neat PBAT and PBAT/curcumin film.

The DSC thermograms of the films are presented in Figure 3. Glass transition was observed around 50 °C in both PBAT and PBAT/curcumin blend films. The similar glass transition temperatures of the neat PBAT and PBAT/curcumin films showed that the blending of curcumin did not pointedly modify the structure of PBAT, indicating curcumin was well-blended with the PBAT polymer matrix.

### 3.3. Surface Color and Optical Properties

**Figure 4:**
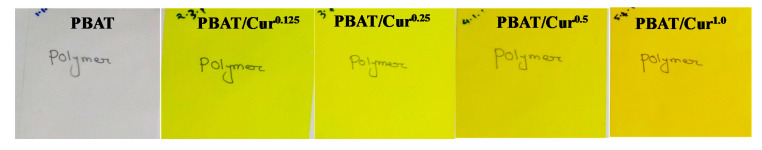
The visual appearance of the neat PBAT and PBAT/curcumin films.

The visual appearance of the PBAT and PBAT/curcumin films are displayed in Figure 4. The neat PBAT film was translucent without any hue but became yellow by the addition of curcumin and became more yellow with increasing curcumin content. The surface color values of the PBAT/curcumin films are presented in Table 2. The *L*-value (brightness) of the PBAT film improved somewhat with the blending of 0.125 wt% curcumin, then decreased linearly with increasing curcumin content. On the other hand, the *a*-value (greenness) decreased significantly with the addition of 0.125 wt% of curcumin and enhanced with increasing curcumin content. However, the *b*-value (yellowness) increased profoundly by the blending of curcumin, indicating a hyperbolic increase pattern according to the curcumin content. Accordingly, the *ΔE* of the films was also enhanced considerably by the blending of curcumin, and it also increased the hyperbolic manner with the curcumin concentration. The *YI* of the PBAT-based film was pointedly enhanced by the blending of curcumin, which was reliant on the curcumin content. Likewise, it was stated that the surface color of the gelatin-based film also transformed greatly depending on the amount of curcumin added [32,33].

**Figure 5:**
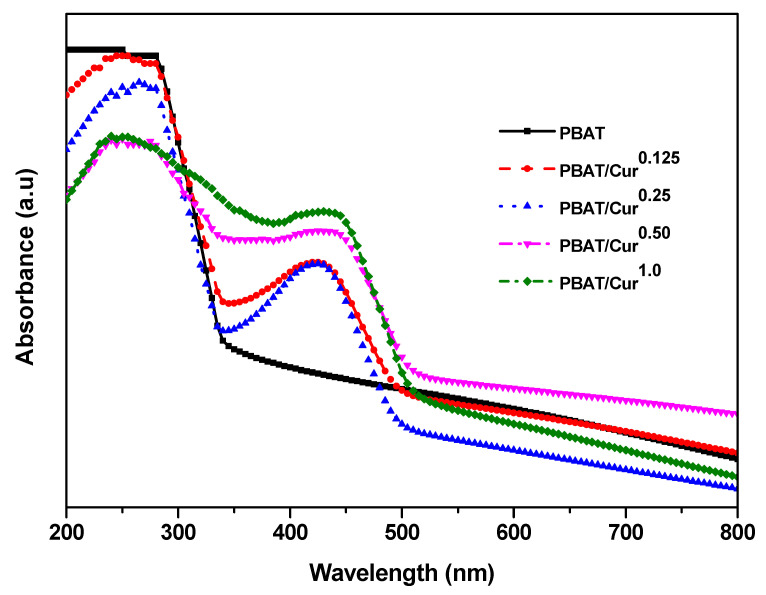
UV-vis light absorption spectra of the neat PBAT and PBAT/curcumin film.

The UV-visible light absorption profile of films are shown in Figure 5. The PBAT film had no visible light absorption peak, but the PBAT/curcumin film exhibited a peak at ~420 nm owing to the presence of curcumin. The peak at low curcumin content (<0.25 wt%) was sharp, but it became broadened at high curcumin content (>0.5 wt%). It was also observed that the light absorption of visible light (>500 nm) of the PBAT/curcumin film were lower or comparable to that of the PBAT film, suggesting that the PBAT/curcumin film is more transparent than the neat PBAT film. Similar light absorption phenomena have been observed in the PBAT/silver nanoparticles films [19]. The PBAT film prevented UV light transmittance nearly completely, which showed the T_280_ of 0.03% (Table 2). It is evident that the PBAT film has high UV-barrier property, which is mainly due to the many UV-light absorbing functional groups in the PBAT [19]. The PBAT film was translucent, having a T_660_ of 5.0%. The transparency of the PBAT film improved a little, which was also evidenced in the light absorption test (Figure 5), but the change was not statistically significant (*p* > 0.05).

### 3.4. Mechanical Properties

The thickness and mechanical behaviors of the films are presented in Table 3. The thickness of the PBAT film increased slightly by the addition of curcumin of less than 0.5 wt%, but it pointedly enhanced (*p* < 0.05) when added a high amount of curcumin (1 wt%). The increased thickness of the film was possibly owing to the enhanced solid content by the addition of curcumin. The mechanical properties of the PBAT film were also influenced by the incorporation of curcumin. The TS of the neat PBAT film was 8.0 ± 0.5 MPa, indicating it is a flexible film with low strength [19]. The TS of the PBAT film decreased a little by the blending of curcumin, which reduced to 6.0 ± 1.1 MPa when 1.0 wt% of curcumin was added. The EB of the neat PBAT film was 23.8% ± 2.3%, representing that the PBAT film is elastic; however, the elasticity of PBAT film reduced a little by the blending of curcumin. The EM (rigidity of the film) of the PBAT film was not changed when a low level of curcumin (0.125 wt%) was added but decreased slightly when more than 0.25 wt% of curcumin was blended. Previously, it was reported that the blending of curcumin did not influence the mechanical behaviors of PBAT films, which might be owing to the compatible and even distribution of curcumin in the polymer matrix, also the appropriate interfacial interactions among them. The present observation was also in compliance with the previously published report [34,45]. Additionally, the blending of curcumin did not change the mechanical properties significantly in the gelatin-based films [32,33]. On the other hand, adding curcumin to the PLA-based film [46] and adding grapefruit seed extract (GSE) to the PLA/PBAT blend film [20] improved the mechanical behaviors of the film.

### 3.5. WVP and WCA

The WVP of the films are presented in Table 3. The WVP of the neat PBAT film was 7.1 ± 0.7 × 10^−11^ g⋅m/m^2^⋅Pa⋅s, which was reduced significantly (*p* < 0.05) by the addition of curcumin. The WVP of the PBAT film decreased to 5.8 ± 0.4 × 10^−11^ g⋅m/m^2^⋅Pa⋅s when 1.0 wt% of curcumin was added. The decreased WVP of the film may be due to the reduced hydrophilicity of the PBAT film through the introduction of hydrophobic curcumin in the PBAT polymer matrix, which hindered the water vapor diffusion and reduced the WVP of the film. The hydrophobic curcumin dispersed in the polymer matrix may reduce water vapor dissolution and diffusion to reduce the WVP. Besides, the rise in the vapor barrier properties of PBAT films with the incorporation of curcumin may be due to an increase in the density of the film or a decrease in free volume after mixing with curcumin (Figure 1). It has been stated that the blending of curcumin to PLA-based film reduced the WVP, whereas the addition of GSE to PLA/PBAT composite film reduced the water vapor barrier properties [20,47]. On the contrary, the addition of impermeable nanoparticles such as silver nanoparticles and graphene oxide was supposed to form a tortuous path of water vapor diffusion in the PBAT film to reduce the WVP [17,19].

The hydrophobicity of the neat PBAT and PBAT/curcumin films was assessed by determining the WCA (Table 3). The WCA of the neat PBAT film was 55.9° ± 2.4°, suggesting that the PBAT film has a water-absorbing surface. The WCA of PBAT film significantly increased (*p* > 0.05) by the blending of curcumin, depending on the content of curcumin. The increase in the WCA of PBAT-based films most probably because of the hydrophobic nature of curcumin [33]. The addition of curcumin to PLA-based film also pointedly enhanced the surface hydrophobicity of the film [47]. Additionally, the improved surface water-resistant activity by incorporating curcumin was described in previous reports [31,34,47].

### 3.6. Release of Curcumin

**Figure 6:**
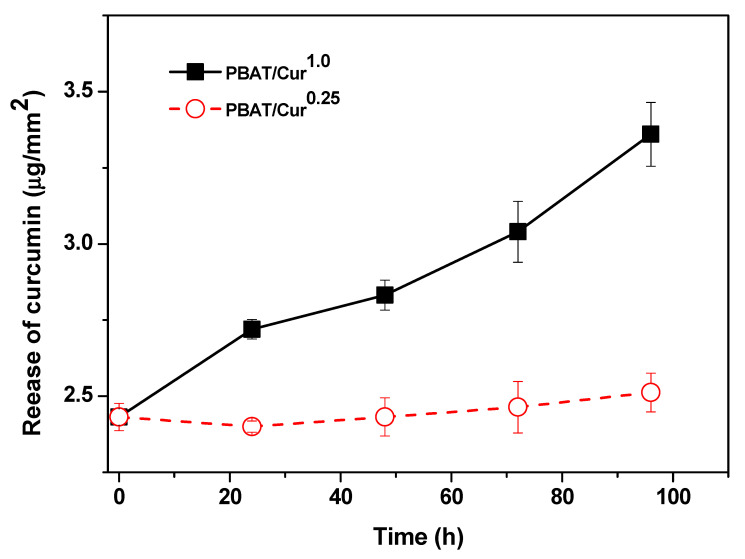
The releasing pattern of curcumin from the PBAT/curcumin film.

The release of curcumin from the PBAT/curcumin film is shown in Figure 6. The rate of release of curcumin was dependent on the concentration of curcumin. However, for a high curcumin content (1.0 wt%), the amount of curcumin released increased linearly with immersion time. The release rate of curcumin depends on the curcumin content and polymer matrix. A more quick release of curcumin has been observed from carbohydrate-based films and guar gum-based films [27,48]. The slow-release of curcumin from the PBAT-based films was due to the low swelling of the polymer matrix in water and low water solubility of curcumin. The degree of release of the bioactive molecule from the film depends on several aspects like the kind of the polymer matrix, swelling of polymers, and interaction between the polymer and the filler as well as the rate of dissolution and diffusion of the compound in the film matrix and the solubility of the compound in the immersion solution [49].

### 3.7. Antimicrobial Activity

**Figure 7:**
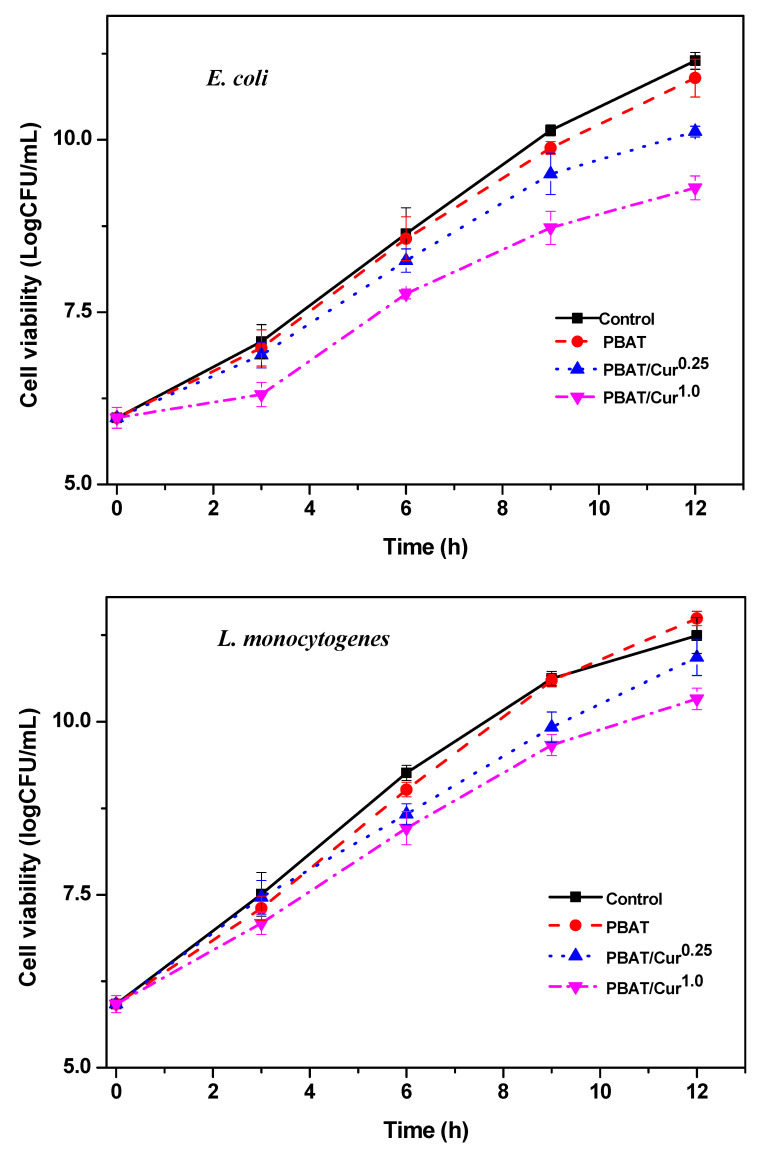
Antibacterial activity of the neat PBAT and PBAT/curcumin films against *E. coli* and *L. monocytogenes*.

As expected, the neat PBAT film showed no antibacterial activity, but the curcumin-added films showed a slight antibacterial activity against both *E. coli* and *L. monocytogenes* (Figure 7). The PBAT/curcumin film did not destroy the bacteria but showed an apparent antibacterial activity that reduces the growth rate of the test bacteria. Depending on the content of curcumin, the film showed a 1–2 log (CFU) lower growth of test bacteria compared to the control groups at 12 h of the test period. Since the neat PBAT film has no antibacterial activity, it can be concluded that the antibacterial function of PBAT/curcumin film is attributed to curcumin [50]. Similar antibacterial activity has been reported for the case of cellulose/curcumin film [34,45]. The small antibacterial activity of the PBAT/curcumin film can be ascribed to the low curcumin content, the slow release of curcumin, and the specificity of the test microbial strain for curcumin. There are reports of high antibacterial activity of curcumin against various fungal and bacterial strains [50]. Conversely, it has been reported that the gelatin/curcumin film does not have a significant antibacterial effect against *S. enteritidis*, *E. coli*, *B. cereus*, and *S. aureus* [32], which may be due to the low curcumin content in the gelatin-based film. Curcumin’s antibacterial activity relies on the interruption of FtsZ role, an essential protein required for bacterial cellular division [51]. The possible antimicrobial activity of curcumin is thought to be because curcumin binds to the FtsZ protein, stopping the joining of the FtsZ protofilament, disrupting the construction of the Z-ring, and impeding cell movement and bacterial development [51]. Curcumin is recognized to increase the GTPase action of the FtsZ protein [52]. It was also believed that the antimicrobial action of curcumin was related with membrane destruction over the binding of the curcumin and peptidoglycan layers of the bacteria [53].

### 3.8. Antioxidant Activity

**Figure 8:**
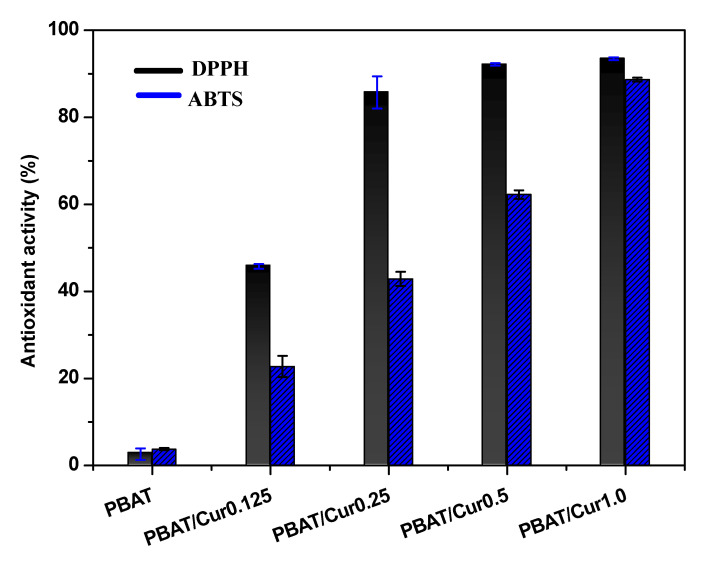
Antioxidant activity of the neat PBAT and PBAT/curcumin films determined by DPPH and ABTS assay.

Because curcumin is a well-known antioxidant [54], the PBAT/curcumin films are also expected to show antioxidant activity. The antioxidant activity of PBAT/curcumin films was evaluated, and the results are shown in Figure 8. As expected, the PBAT/curcumin film showed potent antioxidant activity, and the activity was dependent on the curcumin content. The DPPH and ABTS scavenging activities of the neat PBAT film were 2.6% and 3.8%, respectively, which increased to 93.5% and 88.7%, respectively, after forming the film with 1.0 wt% of curcumin. The high antioxidant activity of curcumin comes from the H-atomic donation function of the curcumin phenol group [54]. The addition of curcumin also provided a strong antioxidant activity for various edible films [32,33,34]. The PBAT/curcumin films can be used as antioxidant packaging for foods that are prone to oxidative degradation.

## 4. Conclusions

The PBAT/curcumin films were fabricated using a solution casting technique. The addition of curcumin affected the surface color, mechanical, water vapor barrier, and antioxidant properties of the PBAT films. The curcumin was uniformly dispersed in the PBAT film and showed a typical light absorption peak at 420 nm. The mechanical properties of the film were slightly decreased after the incorporation of curcumin, whereas the thermal stability of the films was not influenced. The water vapor barrier property of the PBAT/curcumin film was significantly enhanced. The PBAT/curcumin films exhibited high antioxidant activity and some antimicrobial activity against foodborne pathogenic bacteria. The PBAT/curcumin film with increased water vapor barrier and antioxidant activity can be used for food packaging applications to extend the shelflife of food.

## Figures and Tables

**Table 1:** TGA results of the films and curcumin powder.

Sample	*T*_0.5_ (50%) (°C)	Char Content (%)	*T*_max_ (°C)
PBAT	402.6 ± 4.0	7.7 ± 0.9	395.8 ± 3.6
PBAT/Cur^0.125^	404.2 ± 1.4	7.8 ± 0.4	397.7 ± 0.2
PBAT/Cur^1.0^	404.4 ± 0.7	7.8 ± 0.4	397.7 ± 0.2
Curcumin	424.7 ± 6.7	41.0 ± 1.2	381.0 ± 1.3

**Table 2:** Apparent color and light transmittance of the neat PBAT and PBAT/curcumin films.

Films	*L*	*a*	*b*	Δ*E*	*YI*	*T*_280_ (%)	*T*_660_ (%)
PBAT	92.3 ± 0.1 ^b^	−0.29 ± 0.0 ^e^	4.4 ± 0.1 ^a^	0.3 ± 0.1 ^a^	6.9 ± 0.2 ^a^	0.03 ± 0.01 ^a^	5.0 ± 0.8 ^a^
PBAT/Cur^0.125^	93.4 ± 0.4 ^d^	−25.6 ± 0.1 ^a^	74.5 ± 2.4 ^b^	73.6 ± 2.3 ^b^	113.9 ± 4.0 ^b^	0.03 ± 0.01 ^a^	5.7 ± 1.4 ^a^
PBAT/Cur^0.25^	93.2 ± 0.1 ^d^	−23.4 ± 0.1 ^b^	81.9 ± 2.6 ^c^	80.6 ± 2.5 ^c^	125.5 ± 4.0 ^c^	0.04 ± 0.01 ^ab^	5.9 ± 1.7 ^a^
PBAT/Cur^0.5^	92.7± 0.1 ^c^	−21.9 ± 0.9 ^c^	89.9 ± 0.7 ^d^	87.9 ± 0.5 ^d^	138.6 ± 1.2 ^d^	0.06 ± 0.01 ^c^	6.0 ± 1.1 ^a^
PBAT/Cur^1.0^	89.6 ± 0.1 ^a^	−12.5 ± 0.1 ^d^	92.5 ± 0.7 ^d^	88.7 ± 0.7 ^d^	147.5 ± 1.3 ^e^	0.04 ± 0.01 ^ab^	7.1 ± 1.0 ^a^

The values are presented as a mean ± standard deviation. Different letters (such as a, b, etc.) within the same column indicate significant differences (*p* < 0.05) by Duncan’s multiple range test.

**Table 3:** Mechanical properties, water vapor permeability (WVP), and water contact angle (WCA) of the neat PBAT and PBAT/curcumin films.

Films	Thickness (μm)	TS (MPa)	EB (%)	EM (MPa)	WVP (×10^−11^ g⋅m/m^2^⋅Pa⋅s)	WCA (deg.)
PBAT	54.6 ± 4.1 ^a^	8.0 ± 0.5 ^b^	23.8 ± 2.3 ^c^	88.1 ± 2.8 ^c^	7.1 ± 0.7 ^c^	55.9 ± 2.4 ^a^
PBAT/Cur^0.125^	55.6 ± 1.4 ^a^	6.4 ± 0.1 ^a^	17.4 ± 0.8 ^a^	88.9 ± 2.4 ^c^	6.9 ± 0.5 ^ab^	57.7 ± 3.2 ^ab^
PBAT/Cur^0.25^	58.0 ± 1.8 ^a^	6.8 ± 0.3 ^a^	19.9 ± 1.5 ^ab^	81.0 ± 6.1 ^ab^	6.3 ± 0.5 ^ab^	59.5 ± 3.1 ^b^
PBAT/Cur^0.5^	58.9 ± 1.8 ^a^	6.7 ± 0.4 ^a^	22.7 ± 2.3 ^c^	77.2 ± 2.3 ^a^	5.9 ± 0.6 ^a^	63.2 ± 2.0 ^c^
PBAT/Cur^1.0^	64.7 ± 2.4 ^b^	6.0 ± 1.1 ^a^	18.8 ± 0.4 ^a^	82.6 ± 4.0 ^ab^	5.8 ± 0.4 ^a^	63.3 ± 3.6 ^c^

The values are presented as a mean ± standard deviation. Different letters (such as a, b, etc.) within the same column indicate significant differences (*p* < 0.05) by Duncan’s multiple range test.
